# CoDR: Correlation-Based Data Reduction Scheme for Efficient Gathering of Heterogeneous Driving Data

**DOI:** 10.3390/s20061677

**Published:** 2020-03-17

**Authors:** Junho Park, Yoojin Chung, Jongmoo Choi

**Affiliations:** 1 Department of Computer Science and Engineering, Dankook University, Yongin 16890, Korea; heyheygo99@dankook.ac.kr; 2 Division of Computer and Electronic Systems Engineering, Hankuk University of Foreign Studies, Yongin 17035, Korea; chungyj@hufs.ac.kr

**Keywords:** heterogeneous driving data, intelligent data analysis, data reduction, drowsiness detection, correlation, implementation

## Abstract

A variety of deep learning techniques are actively employed for advanced driver assistance systems, which in turn require gathering lots of heterogeneous driving data, such as traffic conditions, driver behavior, vehicle status and location information. However, these different types of driving data easily become more than tens of GB per day, forming a significant hurdle due to the storage and network cost. To address this problem, this paper proposes a novel scheme, called CoDR, which can reduce data volume by considering the correlations among heterogeneous driving data. Among heterogeneous datasets, CoDR first chooses one set as a pivot data. Then, according to the objective of data collection, it identifies data ranges relevant to the objective from the pivot dataset. Finally, it investigates correlations among sets, and reduces data volume by eliminating irrelevant data from not only the pivot set but also other remaining datasets. CoDR gathers four heterogeneous driving datasets: two videos for front view and driver behavior, OBD-II and GPS data. We show that CoDR decreases data volume by up to 91%. We also present diverse analytical results that reveal the correlations among the four datasets, which can be exploited usefully for edge computing to reduce data volume on the spot.

## 1. Introduction

Many advanced driving assistance systems make use of diverse deep learning techniques. For example, FCW (forward collision warning) and LDW (lane departure warning) systems employ deep neural networks and reinforcement learning [1,2]. For analyzing driver behavior, especially for identifying drowsy status, facial-landmark-based learning and steering-wheel-angle-based multi-layer PNN (perception neural network) have been proposed [3,4]. These solutions are extending to more enhanced autonomous driving where perception, control and planning are all conducted using deep learning [5,6].

Deep learning for autonomous driving needs to handle several challenges, and among them, a mountainous challenge is data acquisition, labeling and management [7]. For this purpose, many researches gather various driving data from cameras, OBD (nn-board diagnostics)-II, smartphones and external sensors [8,9,10,11]. Additionally, leading companies such as Tesla, Google and Baidu announced that they collect a large amount of driving data and are willing to release some datasets for autonomous driving studies [12,13]. Besides, the open source community tries to gather driving data to train better models [14,15].

One issue for gathering driving data is its huge volume. One article says that one autonomous car will use 4000 GB of data per a day [16]. Our experience also shows that the front view recording using a smartphone camera generates 16 GB data per a hour. Furthermore, recent autonomous driving solutions utilize heterogeneous driving data [17], which exacerbates the data volume problem, resulting in substantial storage and network bandwidth costs.

To tackle this problem, this paper proposes a new data reduction scheme; we refer to it as CoDR (correlation-based data reduction). Our approach differs from the traditional data reduction approaches, such as deduplication and compression [18,19]. The key idea of CoDR is exploiting the correlations among heterogeneous driving data and reducing irrelevant data, which is determined depending on the data gathering objective.

Specifically, CoDR gathers four heterogeneous driving datasets: two videos, one OBD-II and one GPS data. One video is used for recording front view and the other for recording driver behavior inside a vehicle. In this study, the data gathering objective is a driver drowsiness monitoring and warning. CoDR first chooses the driver behavior video as pivot data and analyzes it using EAR (eye aspect ratio), which is one of the popular mechanisms for detecting drowsy drivers via facial landmarks [20]. The analysis allows CoDR to identify the intervals that have a potential for drowsy driving. These intervals are defined as the relevant data. Then, CoDR investigates the correlations among heterogeneous driving datasets using the timestamp-based synchronization mechanism [21]. Finally, by utilizing the correlations, it reduces data volume by eliminating irrelevant data from both the pivot and remaining datasets.

We implement CoDR using our experimental environment that consists of a smartphone, a Bluetooth-based OBD-II device and an analysis server. Evaluation results reveal that CoDR can reduce data volume by up to 91% with an average of 89%. In addition, we present various observations that show the correlations among heterogeneous driving data. One interesting observation is that the steering wheel angle data can be used as another good indicator for detecting drowsy drivers.

The rest of this paper is organized as follows. In Section 2, we discuss the background and related work of our proposal. Section 3 describes how CoDR works. Implementation details and evaluation results are explained in Section 4 and Section 5, respectively. The discussion section is Section 6. Finally, we summarize conclusions and explain future work in Section 7.

## 2. Related Work

These days, many researchers and organizations collect diverse driving data for ADAS (advanced driving assistant system) and autonomous driving [8,15,22,23,24,25]. The KITTI dataset is one of the most famous heterogeneous driving datasets, consisting of videos, depth data and location information [8]. It provides various benchmarks for stereo matching, visual odometry, object detection, orientation estimation and so on. Chen et al. introduced another driving dataset that supports not only videos and vehicle status, but also large-scale high-quality point clouds scanned by a Velodyne laser [23]. They also demonstrate that the depth information enables one to obtain a comprehensive semantic understanding of real traffic. The BDDV (Berkley DeepDrive Video) dataset provides over 100,000 driving videos with geographic, weather and environmental diversity [24]. In addition, it has a scalable annotation system that can provide a set of image labels for large-scale driving datasets.

These heterogeneous driving data are increasing rapidly in order to enhance analytical accuracy and application domains. However, as the volume of heterogeneous driving data increase, the data acquisition, management and analysis become more and more complex. To address these complexities, new approaches have been proposed in various research areas, including data reduction [26,27,28,29], search space reduction [30] and autonomous data management [31].

Satzoda et al. extracted higher level semantics and events that had the potential to lead to crashes or near-crashes from raw data, and called the dataset NDSs (Naturalistic Driving Studies) [26,27]. Then, they classified the data into different categories which can be utilized effectively by different researchers and accident prevention groups by analyzing a relevant category only. In addition, to made this data reduction automatic, they propose SMZs (safe maneuver zones), which provide a visual tool and quantitative metrics to quantify the threat posed by surrounding vehicles based on their localization in lanes.

Gu et al.’s study [28,29] is closely related to our work. They propose an intelligent DDR (driving data recorder) system that can identify relevant driving data based on the estimated speed and traffic scene understanding. The speed is estimated by the long short-term memory (LSTM) network while the traffic scene is derived by the compressed CNN (convolution neural network). The speed estimation and scene understanding make it feasible to segregate relevant data from irrelevant date, and to reduce data. However, they did not show any quantitative data reduction results.

To the best of our knowledge, this is the first study that considers both the drowsy driver detection and the correlations among heterogeneous data for data reduction, and presents real, implementation-based, quantitative data reduction results.

## 3. Design

The goal of our study is identifying irrelevant data and reducing them to decrease storage and processing overheads for data analysis. Irrelevant data can be defined diversely, according to the objective of data analysis. For instance, assume that we try to investigate the characteristics of aggressive turns from heterogeneous driving data [32]. Then, data ranges gathered while we are driving straightly without turning are irrelevant to this analysis. As an another example, when we analyze FCW (forward collision warning) [2], data ranges that do not contain any obstacles in front of our vehicle are unrelated. When we design our scheme, we are interested in driving conditions such as driver behavior, OBD-II data and front view scenes while driving in a drowsy state. Therefore, the irrelevant data defined in this study are those gathered of a non-drowsy state.

Our proposed CoDR scheme consists of three components; namely, the data collector, the identifier and the manager. The data collector actually gathers driving data using cameras, the OBD-II and the GPS module. The data identifier recognizes relevant data ranges by determining whether a driver is in a drowsy state or not. Finally, the data manager takes care of correlation exploration among heterogeneous driving data and actual data reduction.

**Figure 1:**
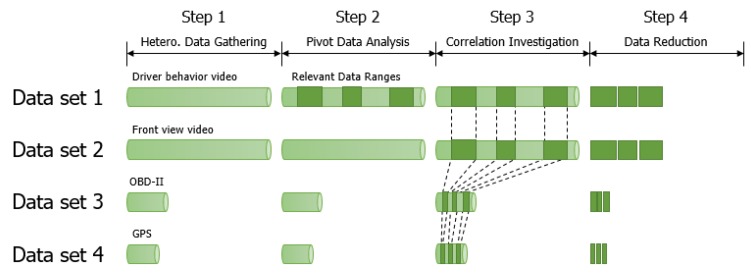
How CoDR works.

Figure 1 illustrates how CoDR works. In the first step, the data collector gathers four different types of heterogeneous driving datasets: (1) a video clip for front view recorded from a built-in camera in a smartphone; (2) another video clip for driver behavior recorded from an external camera; (3) a text file for OBD-II data obtained from a Bluetooth-connected OBD-II device; and (4) a text file for location information from a built-in GPS sensor. The gathered datasets are transferred into the data identifier through WAN or LTE.

Then, from the gathered heterogeneous datasets, the data identifier chooses the driver behavior video as a pivot data and tries to identify data ranges in which one should be suspicious of drowsy driving. Specifically, it locates facial landmarks, such as the eye, mouth and jawline using openface [33] and calculates EAR (eye aspect ratio), which is defined as the following equation:

(1) $$EAR=\frac{\left|\right|p_{2}-p_{6}\left|\right|+\left|\right|p_{3}-p_{5}\left|\right|}{2*||p_{1}-p_{4}\left|\right|}$$

where $p_{1}$, …, $p_{6}$ are the facial landmark locations around an eye [20]. The numerator of this equation is the distance between the vertical eye landmarks, whereas the denominator corresponds to the distance between the horizontal ones.

When EAR is larger than a threshold (0.3 in this study; we will discuss this value further in the evaluation section), it means that a driver has his/her eyes open. Hence, the identifier determines that the driver is in a non-drowsy state. Otherwise, it determines that the driver has the possibility of a drowsy state. The data range wherein EAR is smaller than the threshold is identified as relevant data for analyzing drowsy drivers, as shown in step 2 in Figure 1.

Then, the data manager investigates the correlations among datasets, as illustrated in the step 3 of Figure 1. To that end, CoDR adds a timestamp for each gathered driving datum. For the video, it writes down the first recording time and frame rate. For the OBD-II and GPS data, it logs a timestamp per entry. These timestamps make it possible to synchronize among heterogeneous driving datasets. For instance, it can associate a certain driver behavior with a front view scene and/or an OBD-II entry.

Finally, the data manager reduces data volume by eliminating irrelevant data recognized by the data identifier. It reduces not only the pivot data, driver behavior video in this study, but also the remaining datasets according to the investigated correlations. Besides, it makes an indexing structure, a kind of key-value data structure [34], for fast searching. It uses an offset of a dataset as a key and those of the other datasets as values so that we can find all related heterogeneous driving data directly.

## 4. Implementation

We have implemented CoDR based on a client-server architecture. The data collector can be installed in any client device, including a smartphone, blackbox or embedded board in a vehicle. In this study, we implement it in a Samsung Galaxy S8 smartphone (SM-G950N) that is connected with an OBD-II module via Bluetooth.

**Figure 2:**
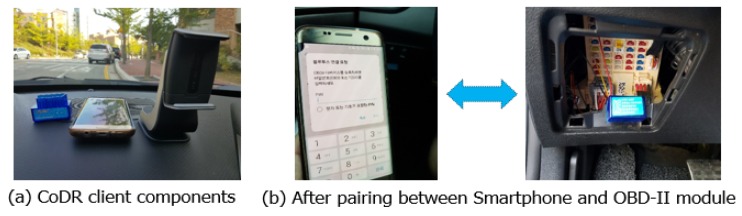
CoDR client: (**a**) main components and (**b**) after completing Bluetooth pairing between smartphone and OBD-II module in a vehicle.

Figure 2 presents key hardware components of our CoDR client, equipped in a car to gather heterogeneous data while driving. In this experiment, we use a Hyundai Grandeur HG 300 and Veloster to gather four datasets: front view video, driver behavior video, OBD-II and GPS data. Each video is recorded at 30 fps (frame per second). The OBD-II data includes various vehicle states, including speed, RPM (rotations per minute), accelerator pedal position, steering wheel angle and brake, which are collected every 100 milliseconds. Finally, GPS data offers location information, such as latitude and longitude, which can be utilized to explore where and in which road conditions a driver tends to be drowsy.

**Figure 3:**
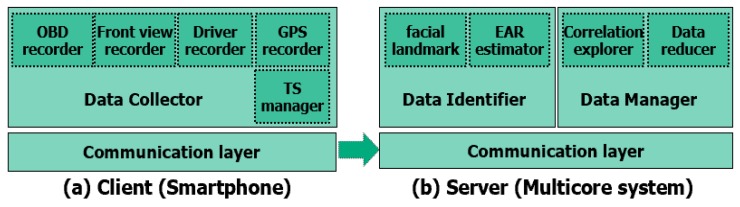
CoDR software architecture.

Figure 3 shows the software architecture of CoDR. It consists of three main components; namely, the data collector, data identifier and data manager. The data collector is implemented in an Android smartphone in a vehicle. It gathers four driving data types: OBD data from an OBD-II device connected via Bluetooth; front view and driver behavior videos using smartphone cameras; and GPS data from an GPS module in the smartphone. It also includes a timestamp managing module (denoted as TS manager in the figure) that adds timestamps for each gathered datum, which will be used for exploring correlations among heterogeneous data.

Both the data identifier and manager are implemented in our experimental multicore system that consists of two Intel XEON processors (six cores per processor), 74GB DDR3 DRAM and Intel 1.2TB NVMe SSD. The data identifier is divided into two modules: one handles the facial landmark detection based on Openface [33] and the other takes care of the EAR calculation using Equation (1) to identify relevant data ranges from the gathered driver behavior video. After finishing this identification, it annotates the relevant data ranges, as shown in the step 2 of Figure 1.

The third component, the data manager, consists of two modules: one is responsible for exploring correlation and the other for data reduction. The correlation explorer makes use of timestamps to correlate heterogeneous data. Specifically, by examining the recorded timestamps in each OBD and GPS data entry and by calculating frame recording times of two videos using the start time and frame rate, we can obtain the correlations among data, as shown in the step 3 of Figure 1. Finally, the data reducer separates relevant and irrelevant data to reduce storage space overhead or to provide relevant data only to the data analysis team.

## 5. Evaluation

In this section, we first explain how much data can be reduced by CoDR. Then, we examine the characteristics observed from our gathered heterogeneous driving data.

### 5.1. Data Reduction Results

**Figure 4:**
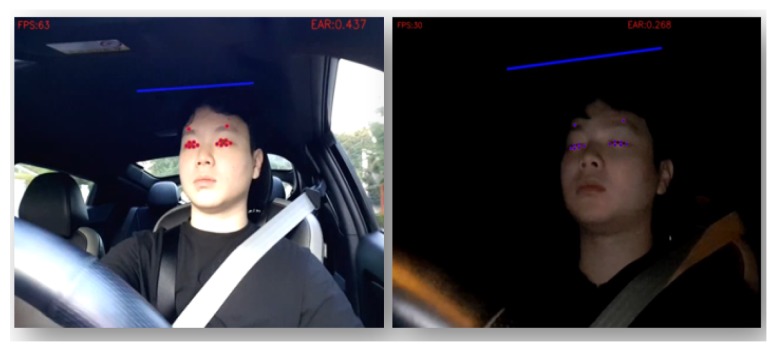
Drowsy driver identification result from the gathered driver behavior video.

Figure 4 shows the drowsy driver identification results. From each frame of the driver behavior video, CoDR locates the eyes using the facial landmark detection mechanism. Then, it calculates EAR (eye aspect ratio); that is, the ratio of the height over width of an eye, illustrated as dotted red circles in the figure. Note that the person in this figure is one of our authors and the circles are generated automatically by the data identifier component in CoDR.

In the left frame of the figure, we can see that when a driver opens his eyes, EAR is calculated as 0.437. On the contrary, in the right frame of the figure, EAR is calculated as 0.208 when the driver closes his eyes. Our experiments exhibit that 0.3 is a reasonable threshold value of EAR for differentiating whether a driver opens or closes his/her eyes. When a driver closes his/her eyes for more than 0.2 s (in other words, when EAR is less than the threshold in six consecutive frames, since we record video at 30 frames per second), CoDR determines that the driver is in a drowsy state and these frames are classified as relevant data.

**Figure 5:**
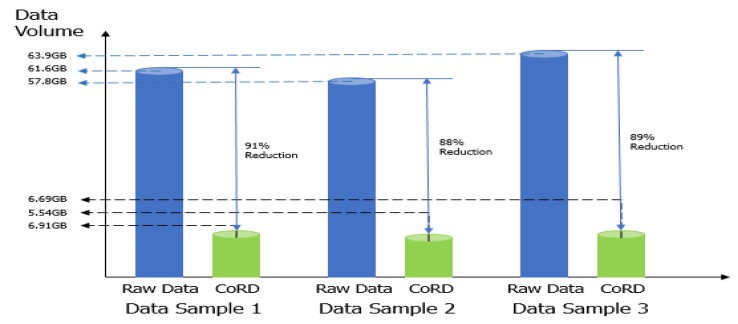
Data reduction result.

Figure 5 presents the data reduction result. CoDR can reduce data volume by up to 91% with an average of 89% by eliminating irrelevant data from not only the driver behavior video, but also other datasets using the investigated correlations. In actuality, CoDR does not eliminate all irrelevant data. It keeps the same size of irrelevant data before and after the identified relevant data to examine the pre and post-conditions of a drowsy state. In addition, CoDR supports an interface so that a user can configure data ranges that he/she wants to store without considering the relevance.

From Figure 5, we can observe that CoDR indeed reduces data volume, providing the opportunity to decrease the storage cost considerably. When we utilize CoDR in an edge computing, it can decrease the network overhead too. In addition, we expect that CoDR can also provide a positive impact on the efficiency of data analysis, since analyzing relevant data only is a kind of search space reduction that can diminish the data processing overhead [26,30].

### 5.2. Correlation Analysis Results

**Figure 6:**
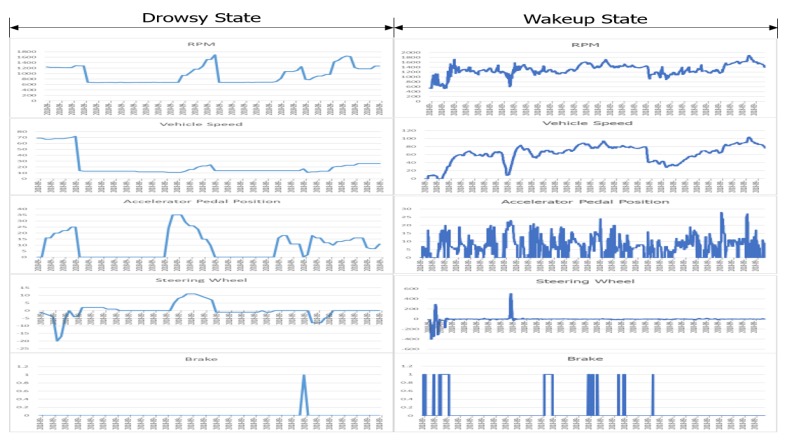
OBD-II data analysis: drowsy state vs. non-drowsy state at a variable speed.

Figure 6 shows the details of the OBD-II data in a drowsy and non-drowsy state, which were identified using EAR from the driver behavior video. We can make the following two observations. The first one is that all gathered OBD-II data items are matched well with each other. For instance, when a driver pushes the accelerator pedal (the third graph), it increases the RPM (the first graph), which in turn increases the vehicle speed (the second graph). Additionally, when the brake is pushed down (the fifth graph), it decreases the RPM, leading to decreased speed. That means that our proposed CoDR gathers the OBD-II data in an appropriate manner.

**Figure 7:**
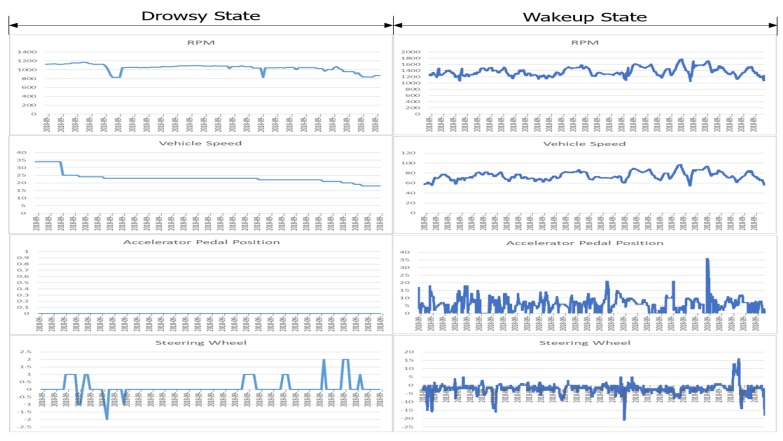
OBD-II data analysis: drowsy state vs. non-drowsy state at a steady speed.

The second observation is that the changes of the OBD-II data in a non-drowsy state are relatively more frequent than those in a drowsy state. The accelerator pedal and RPM are altered ceaselessly in a non-drowsy state. On the contrary, there exists a period where the accelerator pedal and RPM remain unchanged in a drowsy state. At this point, we considered the possibility of a driver driving a car at a steady speed; those results are shown in Figure 7.

Figure 7 presents the OBD-II data when we drive a car at a steady speed in a non-drowsy state. It uncovers that even though we may think we drive cars at steady speeds, there are a number of constant speed changes in a non-drowsy state. On the contrary, there are some periods where the speed remains constant in a drowsy state. One interesting finding is that the steering wheel angle data oscillate repeatedly in a non-drowsy state, whereas they change irregularly in a drowsy state. This implies that the entropy of the steering wheel angle data becomes large in a drowsy state, which can be exploited usefully for detecting sleepy drivers without video data.

One issue related to our study is the sensitivity of our data to different drivers. Our driving data were collected from three different drivers using two vehicles (Hyundai Grandeur and Veloster), whose total distance traveled was around 1500 km. Three samples shown in Figure 5 are chosen from different drivers. They reveal similar data reduction ratios, implying that drivers exhibit homogeneous behavior from the viewpoint of data reduction. However, in terms of other viewpoints, such as the number of sudden brakes or abrupt steering-changes, drivers exhibit different patterns, which was also observed in [35]. We also find that the distinguishable patterns between two states (relatively constant in a drowsy state vs. continuously fluctuating in a non-drowsy state) observed in Figure 7 are stable over time, meaning that they can be used as pertinent indicators for exploring driver drowsiness.

## 6. Discussion

In this section, we first elaborate the main use of our scheme. Then, we discuss how our proposal can be extended in a generalized form.

A deep learning research group is generally divided into two teams; a data acquisition and a data analysis team. The acquisition team takes charge of data gathering, cleansing and management for storage, while the analysis team is responsible for building a learning model, parameter tuning and resource management of computing infrastructure. The acquisition team usually transfers all gathered data to the analysis team. However, these days, as data are collected from multiple sources, the analysis team requests that the acquisition team provide information about correlations among heterogeneous data. Furthermore, as data volume increases rapidly, the analysis team often requests transfers of relevant data only according to their objective. This is where our proposed CoDR is exploited. CoDR is a kind of data preprocessing scheme—also studied in previous research [26,28,30]—which tries to decrease search space prior to actual data analysis.

One issue of CoDR is that the data reduction could affect the data analysis accuracy in metrics such as precision, recall and f-score. In fact, the accuracy depends on not only a learning model and its parameters but also a data sampling method for learning and the amount of noisy data. In terms of noisiness, the data reduction is a double-edged sword. It might give a favorable effect by eliminating a large amount of irrelevant data, but there is a possibility of missing useful data due to the reduction. We expect that the accuracy of data analysis based on the whole data is not noticeably different from that based on the reduced data when we select the same number of positive samples from data ranges in a drowsy state and negatives from data ranges in a non-drowsy state under the uniform distribution. However, the quantitative evaluation is out of the scope of this paper.

Now let us discuss how to extend our scheme in a generalized form. There are four steps in CoDR, as shown in Figure 1, and the second step is a key ingredient for generalization. We design the second step through a generic interface using polymorphism so that we can link an appropriate function dynamically into the generic interface depending on the analysis objective. This paper demonstrates one specific objective; the interface is linked with a function that determines whether a driver has his/her eyes open or closed based on EAR for drowsiness detection. Our scheme can also be used for other objectives, such as over-speed recognition, aggressive turn detection and good driver selection, wherein the generic interface is linked with a function of speed estimation [29], steering wheel angle calculation [32] and driving score assessment [35], respectively.

Our finding about the correlations among heterogeneous driving data can be exploited usefully in various areas. One such area is edge computing. Assume that we want to develop a drowsy driver warning system in a vehicle. Applying the computer vision [20] or deep learning [3] used in this paper to edge computing is challenging, due to the resource limitations of embedded on-board equipment in a vehicle. However, our analysis reveals the correlations between OBD data and driver behavior video. Another area is making use of multiple correlated driving events to enhance analysis accuracy [32,35]. Besides, our approach can be used for data clustering on existing driving datasets, such as KITTI [8] and BDDV [24]. As a future study, we intend to implement a real-time CoDR scheme in an embedded board or a smartphone in a vehicle. Assume that we want to gather dangerous driving data—drowsy driving or aggressive turns, for insurance—for traffic accident investigation purposes. Then, our scheme monitors the steering wheel angle from OBD-II data and differentiates a drowsy and non-drowsy state based on the entropy, as discussed using Figure 7. Finally, it does not save the correlated bulky video data during a non-drowsy state, which eventually decreases the storage and network cost.

## 7. Conclusions

This paper proposes a new data reduction scheme. Unlike the traditional schemes, such as deduplication and compression, it makes use of the identification of relevant data and correlations among heterogeneous datasets. Real implementation-based experimental results have shown that it indeed reduces data volume significantly. Besides, this paper presents various analysis results that disclose the correlations among driver behavior and OBD-II data.

There are three directions for future research. The first one is extending the steering wheel angle data for identifying drowsy drivers using entropy. The second direction is evaluating the effectiveness of the indexing structure quantitatively using an application with multiple windows, one per driving dataset. The final direction is employing edge computing, which can reduce data volume on the spot without transferring data into the server.
